# Experimental Insights into Islanding Detection in PV Inverters: Foundations for a Parallel-Operation Test Standard

**DOI:** 10.3390/s25247582

**Published:** 2025-12-14

**Authors:** Krzysztof Chmielowiec, Aleks Piszczek, Łukasz Topolski

**Affiliations:** 1 Department of Power Electronics and Energy Control Systems, Faculty of Electrical Engineering, Automatics, Computer Science and Biomedical Engineering, AGH University of Science and Technology, 30-059 Kraków, Poland; apiszczek@student.agh.edu.pl; 2 Enersim Sp. z o.o. Armii Krajowej 18, 30-150 Kraków, Poland; lukasz.topolski@enersim.pl

**Keywords:** photovoltaic inverter, islanding detection, grid codes, smart grids

## Abstract

With the rapid increase in photovoltaic (PV) micro-installations in Europe, ensuring the stability and safety of the power grid has become a critical challenge. A key aspect in this context is the reliable detection of unintentional islanding by distributed energy resources. This paper presents the results of metrological tests on seven commercially available three-phase and single-phase PV inverters, conducted in accordance with the requirements of the EN 50549-1 and EN 62116 standards. A dedicated test setup was developed to enable measurements following standardized procedures. The tests assessed both the response time and the effectiveness of islanding detection mechanisms under various fault scenarios, including simulations of autonomous operation of multiple inverters. The main findings indicate that while all inverters with active islanding protection successfully detected islanding within the mandated 2-s limit, their individual response times varied significantly. Parallel operation further influenced this behavior: when one inverter operated with its islanding protection intentionally disabled, the remaining units exhibited notably increased detection times, though still within regulatory thresholds. Moreover, the inverter with disabled protection was capable of sustaining stable islanded operation indefinitely under balanced load conditions. Repeated multi-inverter tests also revealed significant variability in detection time within the same scenario, demonstrating that detection dynamics are sensitive to subtle changes in operating conditions. These findings highlight important limitations of existing certification procedures, which focus primarily on single-inverter testing. Real-world interactions between simultaneously operating inverters can substantially affect detection performance. The results therefore support the need to revise and extend test standards to include controlled multi-inverter parallel-operation conditions, ensuring the safe integration of prosumer PV systems into distribution networks.

## 1. Introduction

The rapid expansion of photovoltaic (PV) micro-installations connected to low-voltage (LV) networks in Poland and across Europe is significantly transforming the structure of modern power systems. The increasing number of prosumers and the growing penetration of distributed energy resources (DERs) introduce new challenges for distribution system operators (DSOs) [1]. A critical aspect of the safe integration of DERs is the ability of PV inverters to rapidly and reliably detect unintentional islanding, which can pose hazards to technical personnel, damage connected loads, and compromise network stability. Compliance with this requirement is mandated by international standards and national grid codes, forming a prerequisite for inverter operation.

This study aims to evaluate the effectiveness of islanding detection in selected commercially available three-phase PV inverters. The experimental investigations were conducted in accordance with the EN 50549-1:2019-02 standard [2], encompassing measurements of both response time and reliability of protection mechanisms under diverse network conditions. Particular emphasis was placed on the influence of variable load-to-generation ratios on detection performance, as well as the resilience of detection algorithms in scenarios where one inverter operated with its islanding detection function disabled. These tests aimed to determine whether, and for how long, inverters could autonomously sustain an island under such conditions.

The primary objective of this research is to conduct a time-response analysis of islanding detection mechanisms, not only for single inverters but also for parallel-connected configurations. This approach is crucial for assessing real-world risks, as the standardized testing of single units may not capture the complex interactions that occur in dense PV installations. The study specifically addresses the critical question of whether a group of inverters can sustain an unintentional island, especially when one device in the network has its protection intentionally or faultily disabled, and quantifies the resulting impact on overall detection times. The analysis encompasses both three-phase and single-phase PV inverters.

According to EN 62116 [3], which specifies procedures for verifying islanding detection methods, unintentional islanding occurs when one or more distributed generators continue to supply an isolated portion of the network following its disconnection from the main grid. This phenomenon represents a significant metrological and operational challenge, requiring precise measurement of response time, detection effectiveness, and repeatability of protective actions. Inadequate or delayed detection of islanding can compromise distribution system safety, both from technical and operational perspectives.

Unintentional islanding presents several risks: it endangers technical personnel performing maintenance or repair tasks who may be unaware of the presence of voltage in an isolated network segment; it can degrade power quality by causing voltage and frequency instability, adversely affecting connected loads; and it may induce unexpected power flows and disturbances in the local distribution network.

The paper is structured as follows: Section 2 provides a state-of-the-art review, categorizing and analyzing existing islanding detection methods and discusses the impact of advanced inverter control strategies on detection reliability. Section 3 details the methodology, describing the dedicated laboratory test setup, the metrological procedure based on the EN 50549-1 standard, and the definition of the Non-Detection Zone (NDZ) used for testing. Section 4 presents the use case and results, comparing islanding detection times for individual inverters and parallel configurations, including scenarios with a disabled protection unit. Section 5 introduces a proposed framework for extending existing standards to multi-inverter conditions, based on insights gained from the experiments. Section 6 concludes the paper by summarizing the key findings, while Section 7 outlines potential future research directions to further enhance islanding detection in evolving power networks.

## 2. State-of-the-Art

### 2.1. Islanding Detection Methods

Islanding detection methods are critical in distributed generation (DG) systems, particularly those integrated with renewable sources like PV and wind, to ensure safety, stability, and compliance with grid codes. Islanding occurs when a DG unit continues supplying power to a local load after disconnection from the utility grid, potentially leading to hazards such as out-of-phase reclosing, equipment damage, and risks to utility workers. This review synthesizes key advancements in islanding detection methods, categorizing them into passive, active, communication-based (remote), modified passive (signal processing-enhanced), intelligent, and hybrid approaches. The discussion draws from comprehensive surveys, highlighting principles, techniques, advantages, disadvantages, and performance metrics such as NDZ, detection time, and reliability.

Modern photovoltaic inverters most commonly implement islanding detection through a hybrid of active and passive methods. Passive approaches involve the continuous monitoring of network parameters, including voltage, frequency, and their respective derivatives, whereas active approaches deliberately introduce perturbations into the system and subsequently assess the corresponding network response. The combined implementation of these strategies enhances detection reliability while simultaneously preserving operational stability under nominal grid conditions.

#### 2.1.1. Passive Methods

Passive islanding detection methods monitor inherent system parameters (e.g., voltage, frequency, harmonics) at the point of common coupling (PCC) without perturbing the system, relying on natural deviations post-islanding. Key techniques include:

- Over/under voltage/frequency protection (O/UVP, O/UFP): Threshold-based relays trip on deviations (e.g., voltage ± 10%, frequency ± 0.5 Hz) [4].
- Rate of change of frequency/power (ROCOF/ROCOP): Detects rapid changes (df/dt > 0.1 Hz/s) due to power imbalance [5].
- Phase jump detection: Identifies sudden voltage–current phase shifts [6].
- Harmonic monitoring: Tracks total harmonic distortion (THD) increases [7].
- Phasor-based method [8].

Passive methods offer several advantages, including their simplicity, low cost, lack of power quality degradation, and straightforward integration with existing relays. However, they suffer from significant drawbacks, such as a large NDZ for balanced loads where power mismatches (ΔP ≈ 0, ΔQ ≈ 0) are minimal, as well as a susceptibility to false trips triggered by grid transients. In terms of performance, these methods exhibit a NDZ of up to 20–30% power mismatch, detection times ranging from 2–6 cycles (33–100 ms at 60 Hz), and reliability of 70–80% under ideal conditions, with early evaluations underscoring their limitations in PV systems featuring closely matched generation and load scenarios.

#### 2.1.2. Active Methods

Active islanding detection methods operate on a principle of controlled provocation. They function by having the inverter introduce deliberate disturbances into the electrical system it is powering. The core logic is that the presence or absence of the main utility grid will dictate the system’s response to these disturbances, thereby revealing its true state.

When connected to the utility grid, the grid acts as an immense, stabilizing force. It possesses such overwhelming inertia and voltage regulation that any small disturbance injected by a single inverter is instantly absorbed and corrected, leaving no measurable trace. The grid’s frequency is rigid, and its voltage is steadfast. However, in an islanded condition, where the grid is disconnected but local power generation matches local power consumption, a delicate, standalone microgrid is formed. In this isolated state, the inverter and the local load are the only players. The small disturbances from the inverter are no longer swallowed by the grid’s vastness. Instead, they interact directly with the local load’s characteristics. If the load is not a perfect match, these minor perturbations can be amplified, leading to a measurable and growing deviation in voltage or frequency.

AFD [9] is a foundational technique that embodies this concept. It works by distorting the output current waveform of the inverter. The inverter intentionally inserts a brief pause, or zero-crossing delay, into its current waveform, making it slightly out of phase with the voltage. In grid-connected mode, the grid’s perfect sinusoidal voltage waveform forces the current to conform, rendering this distortion ineffective. But in an islanded state, the inverter itself dictates the voltage waveform. This intentional phase shift accumulates with each cycle, acting like a gentle but persistent push on the system’s frequency, slowly driving it upwards or downwards until it drifts beyond the standard protection thresholds and triggers a shutdown. Article [10] presents a modified AFD method for detecting unintentional islanding in inverters supplying renewable energy to the grid. The objective of islanding detection methods is to minimize the duration of power supply to the isolated grid section. The proposed method eliminates constant segments of the reference current signal, replacing them with a hyperbolic sine function, which reduces total harmonic distortion (THD) while maintaining detection effectiveness. The approach details a new disturbance generation method and was validated through both simulation and laboratory tests, confirming the potential to shorten islanding detection time by increasing maximum current distortion without exceeding permissible THD limits for grid-connected inverters.

Building upon this, the Sandia Frequency Shift method [11] introduces a more aggressive and intelligent strategy: positive feedback. It does not just apply a fixed distortion but actively listens to the system frequency. If it detects even a minuscule deviation—a tiny dip or rise, according to Equation (1)—it does not just note it; it reacts by commanding a larger current distortion (2).

(1) $$\theta_{SFS}=k_{f}(f_{PCC}-f_{nom})$$

(2) $$i\left(t\right)=I_{m}sin(2\pi ft+\theta_{SFS})$$

where θ_SFS_ is the additional phase shift (in radians or degrees) applied to the current waveform, k_f_ is the frequency feedback gain (a carefully chosen constant >0), f_PCC_ is the measured instantaneous frequency at the PCC, and f_nom_ is the nominal grid frequency (50 Hz or 60 Hz). This larger distortion pushes the frequency further in the same direction, which the algorithm then detects and responds to with an even stronger push. This creates a runaway effect, a positive feedback loop that rapidly accelerates any initial frequency deviation, ensuring a swift and decisive trip in an islanded condition. It is a method designed to force an unstable condition where one would not naturally occur.

Similarly, the Sandia Voltage Shift (SVS) [12] applies the same positive feedback principle, but to voltage. When the inverter senses a slight voltage dip, its control algorithm deliberately reduces its output power according to Equation (3):

(3) $$P_{out}=P_{set}[1+k_{p}\left(V_{PCC}-V_{nom}\right)]$$

where P_out_ is the instantaneous active power output of the inverter, P_set_ is the nominal active power setpoint (e.g., the maximum power point tracking output), k_p_ is the positive feedback gain (a carefully chosen constant >0), V_PCC_ is the measured RMS voltage at the PCC, and V_nom_ is the nominal RMS grid voltage.

In a grid-connected scenario, this reduction is negligible and has no effect on the robust grid voltage. In an island, however, reducing power into a constant load will cause a further, more significant voltage drop. The algorithm detects this new, lower voltage and reduces power again, creating another amplifying feedback loop that swiftly drives the voltage down to a level that triggers under-voltage protection.

Other sophisticated approaches include impedance measurement. This method works by having the inverter inject a small, high-frequency current harmonic or a transient pulse into the system and then meticulously observe the resulting voltage response [13]. The ratio of the voltage change to the current change reveals the system impedance. The utility grid presents an extremely low impedance path. A sudden, large increase in measured impedance is a clear signature that the low-impedance grid path is gone, indicating an island.

Active islanding detection methods offer advantages including a smaller NDZ often below 5% power mismatch, fast detection capabilities, and strong effectiveness for inverter-based distributed generations. However, they introduce disadvantages such as harmonics and flicker that degrade power quality, dilution effects in multi-DG setups where disturbances cancel out, and the need for complex tuning. In terms of performance metrics, active methods achieve a NDZ of 0–10%, detection times ranging from 0.5 to 2 s, and reliability of 80–90%.

#### 2.1.3. Hybrid Methods

Hybrid islanding detection strategies are engineered to leverage the complementary strengths of both passive and active methods [14,15]. These approaches are typically implemented in a two-stage sequence to optimize both speed and reliability. The process initiates with a passive monitoring stage, which continuously observes native grid parameters like voltage, frequency, and harmonic distortion. This first stage acts as a high-speed, low-impact screening tool, designed to rapidly flag a potential islanding event based on pre-set thresholds. For example, ref. [16] introduced an innovative hybrid method using rate of change of voltage (ROCOV) analysis. Tests on an EMT (Electromagnetic Transient) model demonstrated very good effectiveness while maintaining high selectivity against asymmetric faults, sudden load changes, and capacitor bank switching.

#### 2.1.4. Communication-Based (Remote) Methods

These methods represent a fundamentally different approach from local techniques by leveraging the direct communication link between the utility and DG sources. Instead of relying on the indirect inference of the grid’s status by monitoring local electrical parameters like voltage or frequency, these methods use a dedicated channel for explicit monitoring or signaling. This allows them to bypass the inherent limitations and NDZ associated with local measurements. Key implementations of this strategy include power line carrier communication [17], where the utility injects a continuous sub-harmonic signal onto the grid that is monitored by the DG—the loss of this signal immediately triggers a trip command. Another method utilizes transfer trip schemes [18], which involve dedicated communication lines, such as fiber optics or wireless networks, to send a direct disconnection signal from the utility to the DG upon the opening of a key circuit breaker. Furthermore, broader supervisory control and data acquisition (SCADA) systems can be employed for centralized, real-time monitoring of breaker status and system-wide parameters to coordinate anti-islanding protection.

The principal advantage of these communication-based schemes is their exceptional performance: they boast a nearly non-existent NDZ, offer extremely fast detection times often under one second, and are highly reliable with metrics typically exceeding 95%, making them scalable and ideal for complex networks like microgrids. However, these benefits come with significant trade-offs, primarily very high infrastructure costs for installing and maintaining the communication hardware and channels. Their performance is also entirely dependent on the integrity of the communication link, meaning any failure in the signal path can lead to a failure to detect an island. Furthermore, they require a high degree of utility cooperation and integration. Consequently, while these methods offer a gold standard in performance for large-scale or critical applications, their cost and complexity often render them uneconomical for small-scale, residential photovoltaic systems.

#### 2.1.5. Intelligent Methods

Intelligent islanding detection methods represent a paradigm shift from conventional techniques by moving away from rigid, pre-defined thresholds and instead employing sophisticated computational intelligence to analyze the complex state of the electrical network. These methods leverage machine learning and pattern recognition to process a multitude of extracted features from voltage and current signals, enabling them to adapt to dynamic grid conditions and distinguish islanding events from other similar disturbances, such as capacitor switching or large motor starts, with high precision. The core of this approach lies in its data-driven nature. Instead of being programmed with a fixed logic, these algorithms are “trained” on a comprehensive dataset that includes examples of both grid-connected and islanded operation, along with various other common grid disturbances. This training allows them to learn the subtle, often non-linear patterns that characterize an islanding event. Key techniques embody this principle in different ways. Artificial neural networks, particularly probabilistic neural networks, function as powerful non-linear classifiers [19,20]. They are trained to analyze input features—such as the harmonic spectrum of voltage, rate of change of frequency, or phase angle displacement—and map them to a probabilistic output, classifying the system state as “islanded” or “grid-connected.”

Support vector machines operate on a different principle, seeking to find an optimal hyperplane that maximally separates the data points representing different classes of events in a high-dimensional feature space [21,22]. Their strength lies in their ability to perform this separation effectively even with a relatively small number of training samples.

Furthermore, fuzzy logic systems provide a robust framework for handling the inherent uncertainty and imprecision in electrical measurements [23,24]. Unlike binary logic, fuzzy logic uses linguistic variables and a degree of membership, allowing it to implement expert knowledge through rules such as “IF the voltage deviation is slightly low AND the frequency change is moderately high, THEN the islanding probability is high.” This makes them exceptionally resilient to noisy data and parameter variations.

The primary advantage of these intelligent methods is their exceptional performance in complex, modern grid environments. They achieve high accuracy even in networks with multiple distributed generators and significant background noise, precisely because they learn from the complex interactions within the system. Crucially, their NDZ is not dependent on the careful setting of fixed thresholds, as with active methods, but rather on the quality and comprehensiveness of their training. However, these advantages come with distinct challenges. The performance of these systems is heavily dependent on the availability of extensive and representative training data that covers a wide spectrum of possible grid scenarios. They can also be computationally intensive during the training phase, though once trained, the execution for detection is typically fast enough for real-time operation. There is also a persistent risk of overfitting, where a model becomes overly specialized to its training data and fails to generalize to unseen, real-world events. In terms of performance, these methods are capable of achieving detection times under half a second with accuracy rates ranging from 90% to 99%, effectively reducing the NDZ to less than 5%. Promising research is now focused on hybrid models, such as ANN-SVM combinations, which leverage the strengths of different algorithms to create more robust and efficient systems, making them increasingly viable for real-time integration in photovoltaic and other distributed energy systems.

### 2.2. Operational Requirements for PV Inverters in Distribution Networks

**Figure 1:**
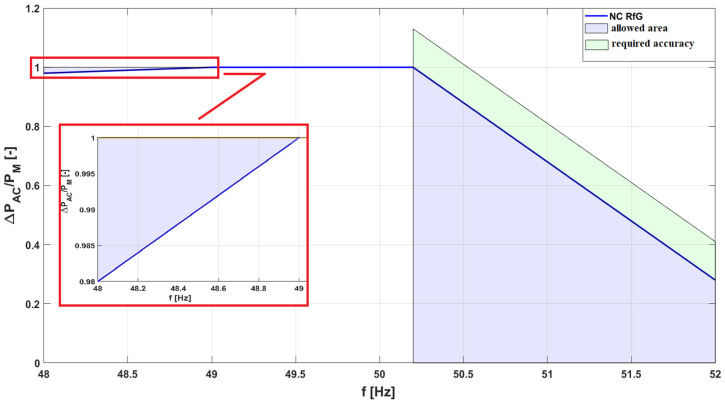
The permissible operating range of PV inverters when changing the PV inverters against AC voltage frequency of the supply voltage.

**Figure 2:**
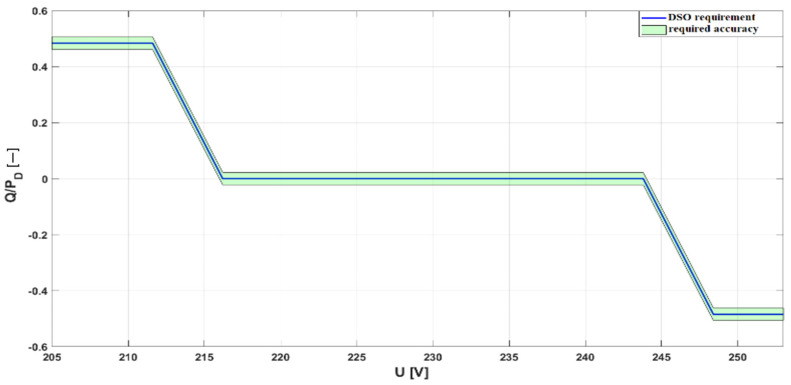
The required operating range of photovoltaic inverters in the reactive power control mode.

**Figure 3:**
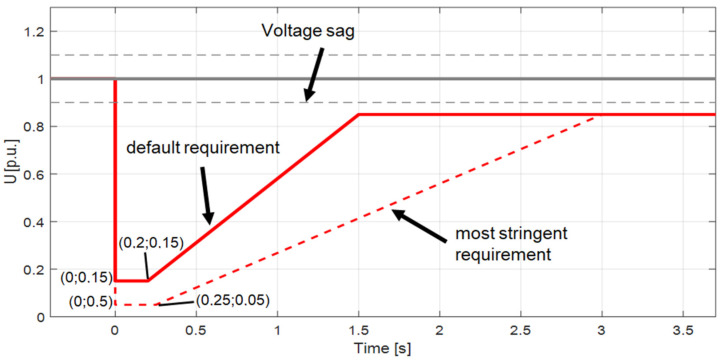
Under-Voltage Ride Through capability recommendation for type A modules.

The PV inverter, serving as the core component of any PV micro-installation, is responsible for converting direct current (DC) into alternating current (AC). Its operation requires careful consideration, as its design and performance directly impact power system safety, installation reliability, and power quality at the LV point of connection. To ensure proper inverter operation, DSOs have implemented a set of requirements aimed, among other objectives, at limiting voltage rise caused by energy generation. These requirements are specified in local DSO network codes [25]. According to these documents, PV inverters must meet the following control mode requirements:

1. Overfrequency power limitation (LFSM-O—Limit Frequency Sensitive Mode)—requiring active power reduction when the frequency exceeds 50.2 Hz, with a programmable gradient of 2% (Figure 1).
2. Reactive power control, e.g., via Q (U) or cosφ (P) characteristics (Figure 2).
3. Active power control (P(U))—activated upon reaching the maximum reactive power regulation capacity.
4. Under-Voltage Ride Through (UVRT) and Over-Voltage Ride Through (OVRT)—ensuring inverter resilience to defined voltage-time profiles (required for Type A generators, Figure 3)
5. Islanding detection—implemented via passive methods (e.g., ROCOF—Rate of Change of Frequency) or active methods (e.g., active frequency drift).

Modern PV systems demand advanced control strategies that go beyond basic regulatory requirements to enable optimal integration with distribution networks. The literature shows significant progress in control algorithms designed to enhance grid stability. Proper technical assessments (grid-codes) are essential to ensure any renewables’ connection point meets required voltage, asymmetry, and distortion limits as generation increases [25,26,27,28].

In papers [29,30] highlight significant discrepancies in how prosumer-level photovoltaic inverters available on the market meet regulatory requirements. They show that while many devices formally comply with European network codes, their performance often falls short of stricter national distribution system operator standards. The studies emphasize the need for more rigorous testing, transparent settings, and stronger verification procedures to ensure reliable grid integration.

Analyzing the available literature in the considered field, the review in [12] provides a comprehensive assessment of islanding detection techniques, including frequency shift methods, voltage shift methods, frequency variation, phase jump methods, and active and reactive power-based approaches, evaluating them in terms of reliability, sensitivity, complexity, cost, and compliance with IEEE standards. The study in [31] proposes new detection algorithms based on the rate of change of voltage imbalance, improving performance compared to traditional passive and active methods, particularly in networks with PV systems. Similarly, ref. [32] presents a review of methods for PV inverters, classifying them as passive, active, or remote, with particular attention to detection-limited zones, highlighting the challenges of minimizing such zones under variable load conditions. In ref. [33], a PV inverter model with islanding detection is introduced, demonstrating through MATLAB/Simulink simulations how current phase angle perturbations with positive feedback trigger frequency protection. The article in [34] focuses on techniques for inverter-based distributed generators, classifying methods as passive (e.g., voltage imbalance, harmonic distortion) and active, emphasizing their effectiveness in PV systems, albeit with potential impacts on power quality, and proposing hybrid approaches for improved accuracy. Overall, the analysis of these works indicates an evolution from passive to hybrid and intelligent methods, with a need to enhance reliability under conditions of high renewable energy penetration.

In the area of inverter testing under multi-inverter scenarios, report [35] presents a critical experimental evaluation of an emerging safety risk in solar-rich power grids. The study focuses on how mandatory grid-support functions in modern photovoltaic (PV) inverters can inadvertently interfere with their essential anti-islanding protection. The core finding is that, in a multi-inverter island scenario, the inverters’ collective attempts to regulate voltage and frequency can stabilize the isolated grid segment. This stabilization prevents the individual inverters from detecting the grid outage, as their protection schemes rely on observing such disturbances. Consequently, the report provides clear evidence that existing anti-islanding test standards—often based on single-inverter evaluations without grid-support functions enabled—are no longer sufficient. It highlights the urgent need to update safety certification protocols to reflect these complex, interactive behaviors observed in real-world multi-inverter systems, in order to prevent dangerous islanding conditions.

In addition, numerous simulation-based studies on islanding detection are available in the literature [36,37]; however, none of them specifically address the operation of inverters in a multi-inverter scenario where anti-islanding detection is intentionally disabled in some of the inverters.

### 2.3. Impact of Different PV Inverters Control Strategies on Islanding Detection

Islanding detection relies on measuring changes in grid parameters (voltage, frequency, power) at the PCC. When the grid disconnects, these parameters are supposed to shift beyond normal limits, triggering the inverter’s protection relays. However, advanced grid-support functions, mandated by modern grid, are designed to prevent these very changes. They actively control the inverter’s output to stabilize the grid, which inadvertently makes an island harder to detect.

Active power control, specifically the active power–frequency (P–f) strategy, programs the inverter to adjust its real power output in response to grid frequency deviations, such as reducing power during over-frequency events and increasing it during under-frequency events if capacity allows. This frequency–watt droop control, while crucial for grid stability, has a severely negative impact on passive islanding detection. The fundamental conflict arises because during an islanding event, any load-generation imbalance naturally causes a frequency shift. The P–f control interprets this shift as a grid anomaly it must correct, actively working to neutralize the frequency drift by modulating power output. Consequently, it suppresses the very frequency trigger that passive protection relays rely on to detect the island, potentially creating a stable but undetected island. This inherent limitation forces a reliance on active islanding detection methods, which must inject a deliberate and significant disturbance to overcome the inverter’s own stabilizing efforts and ensure the island is recognized and de-energized.

Reactive power control (Q–V) strategy, directs the inverter to modulate its reactive power output to stabilize grid voltage, for instance, by consuming reactive power during voltage rises and injecting it during voltage sags. This voltage–VAR droop function is profoundly detrimental to passive islanding detection, creating an effect similar to that of active power control. In an islanded condition, the voltage naturally begins to shift due to a mismatch between local generation and load. The Q–V control interprets this change as a disturbance it must correct, automatically adjusting its reactive power output to force the voltage back toward its nominal value. This active compensation effectively masks the islanding condition by preventing the voltage from drifting beyond the set thresholds of the Over/Under Voltage Protection relays. As a direct result, this control strategy significantly expands the non-detection zone, rendering passive methods unreliable.

## 3. Methodology

The primary aim of this study was to evaluate the capability of PV inverters to detect and respond to islanding conditions. According to the standard [3], compliance tests must be conducted at precisely defined operating points characterized by specific generation conditions and circuit imbalances in terms of active and reactive power. These requirements ensure that the system can reliably detect islanding under a variety of realistic operating scenarios.

As part of the certification process, the standard mandates detailed tests including the measurement of the inverter disconnection time in response to a simulated grid disconnection. Specifically, each PV inverter must automatically disconnect within 2 s of islanding occurrence. In the case of detection methods based on the ROCOF, the response time must be reduced to below 500 ms. The standard [3] further specifies that tests should be performed on a single inverter connected to a test grid, enabling assessment under idealized and repeatable conditions. However, this raises questions regarding the applicability of such results to real-world conditions, where multiple PV inverters may be connected in close proximity within the same network area.

To address this, extended tests involving the simultaneous operation of multiple PV inverters are needed. These tests allow the analysis of interactions between units and assessment of whether parallel operation reduces the effectiveness of islanding detection. Additionally, they provide insights into measurement uncertainty and repeatability under more realistic conditions, verifying whether dynamic interactions could inadvertently sustain islanding despite normative requirements.

**Figure 4:**
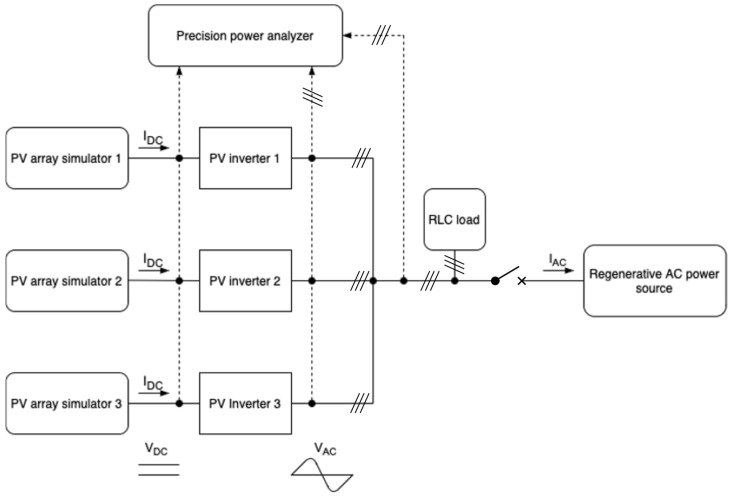
Laboratory test stand for island detection verification.

A specialized laboratory setup, symbolically illustrated in Figure 4 for a three-inverter case, was employed for the experimental investigation. It was designed to enable testing in accordance with normative requirements. The setup was designed to meet normative testing requirements and comprises several sections typical for evaluating low-frequency power quality disturbances [38]. The PV inverters were powered by independent photovoltaic source simulators, implemented using DC amplifiers controlled by signals from a real-time hardware-in-the-loop (HIL) simulator. This setup allowed faithful reproduction of the electrical characteristics of real PV modules across a wide range of operating conditions, with a signal generation error not exceeding 1%.

Each inverter was connected to a common grid simulator, allowing precise regulation and stabilization of key system parameters, including RMS voltage (signal error: 0.1% + 0.2% of full scale) and frequency (signal error: 0.1% + 0.2% of full scale). This enabled replication of both nominal grid operating conditions and disturbance states, which were essential for testing in accordance with the relevant procedures.

To facilitate comprehensive analysis of system behavior under varying loads, passive, adjustable R (resistive), L (inductive), and C (capacitive) loads were installed. Their parameters were selected to allow precise shaping of active and reactive power balances and to control dynamic conditions within the test system. This configuration enabled assessment of the impact of diverse loading scenarios on the response time and effectiveness of islanding detection mechanisms. Instantaneous measurements were conducted using a precision power analysis system, providing power measurement accuracy (at a power factor of 1) of ± (0.01% of reading + 0.02% of range) for AC and ± (0.02% of reading + 0.05% of range) for DC.

### Non-Detection Zone

Test scenarios with balanced variations in active and reactive power (ΔP and ΔQ) were conducted using the regulatory capabilities of the R-L-C load set. The power deviations were calculated as:

(4) $$\Delta P=P_{AC}-P_{load}$$

(5) $$\Delta Q=Q_{AC}-Q_{load}$$

where P_AC_ and Q_AC_ denote the inverter’s active and reactive power output, and P_load_ and Q_load_ refer to the corresponding active and reactive load powers. For the presented tests, the conditions were stabilized such that the absolute imbalances were maintained within a tolerance of ±1% of the inverter’s rated powers:

(6) $$\left|\Delta P\right|\leq 0.01\cdot P_{rated}\;\mathrm{and}\;\left|\Delta Q\right|\leq 0.01\cdot Q_{rated}$$

The study utilized four three-phase and three single-phase prosumer inverters. The three-phase units had similar rated powers (5–6 kW), while the single-phase units were approximately 3.5 kW. Comprehensive, time-synchronized measurements of DC and AC-side currents and voltages were taken at the PCC. This setup enabled analysis of interactions between the devices and the precise determination of islanding protection response times.

The fundamental condition for a stable, undetected island is a minimal power imbalance. The initial active and reactive power imbalance at the instant of grid disconnection (t_0_) is given by (7) and (8). In an islanded condition, the system frequency and voltage at the PCC are governed by the load characteristics and the inverter’s output. The natural drift of these parameters can be modeled by their rate of change. The ROCOF is a critical metric for passive detection:

(7) $$ROCOF=\frac{df}{dt}\approx \frac{∆f}{∆t}$$

where Δf is the frequency deviation from nominal (50 Hz) over a time interval Δt. Similarly, the ROCOV is defined as:

(8) $$ROCOV=\frac{dV}{dt}\approx \frac{∆V}{∆t}$$

A protection relay triggers when these values exceed predefined thresholds (ROCOF_set_, ROCOV_set_).

## 4. Use Case and Results

Table 1 presents the measured islanding detection performance of three-phase photovoltaic inverters operating under various configurations, with specified generated and consumed active and reactive power imbalances. In the first series of tests (tests 1–4), each available inverter was tested individually. Inverters 1, 2, and 3 had anti-islanding protection enabled, whereas Inverter 4 was tested with this protection disabled.

**Figure 5:**
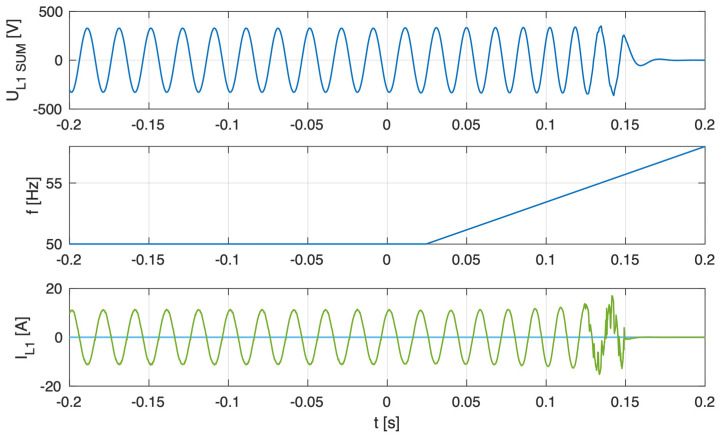
Islanding Detection of the Standalone Operation of Inverter No. 1 (Test 1).

**Figure 6:**
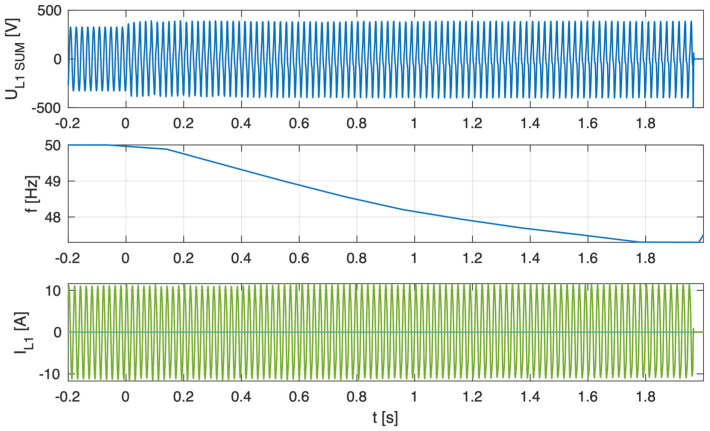
Islanding Detection of the Standalone Operation of Inverter No. 2 (Test 2).

**Figure 7:**
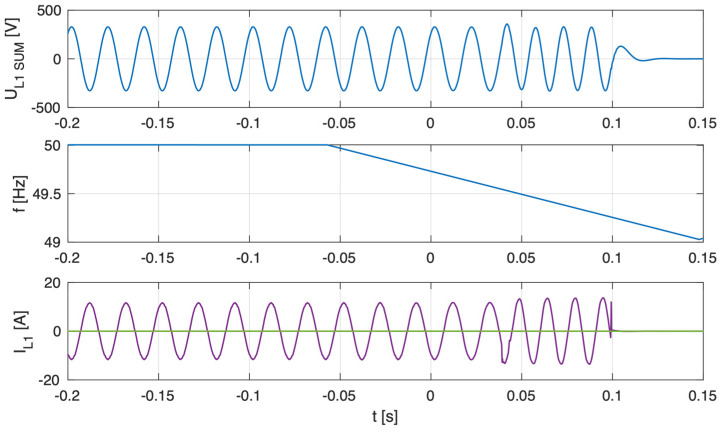
Islanding Detection of the Standalone Operation of Inverter No. 3 (Test 3).

**Figure 8:**
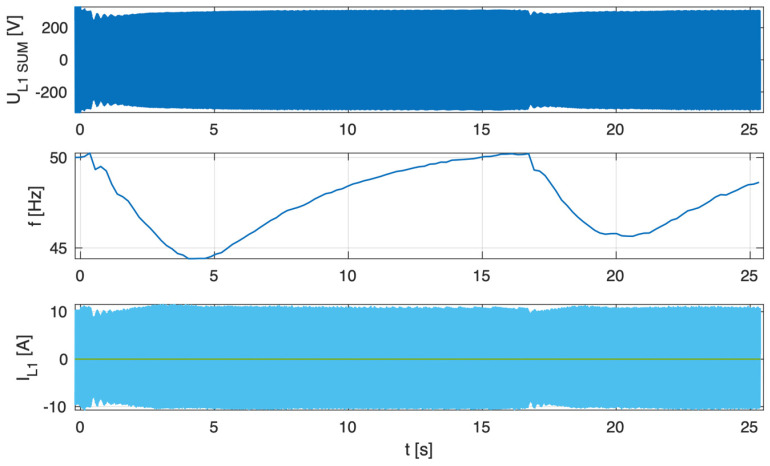
Islanding Detection of the Standalone Operation of Inverter No. 4 (Test 4).

As illustrated in Figure 5, Figure 6, Figure 7 and Figure 8, which depict the autonomous operation of the inverters following the disconnection of the voltage source, inverters with active islanding detection protection disconnected within 280 ms. The exception was Inverter 4 (Figure 8), which, as expected, did not disconnect and sustained autonomous operation indefinitely while the load-generation balance was maintained.

Inverter 4 was also tested under unbalanced conditions, with the parameter ΔP set to +5% Pac and −5% Pac. In the first case (Test 5), the inverter disconnected due to an excess of generated power, which exceeded the load’s ability to consume it. In the second case (Test 6), the PV system generated insufficient power. However, this did not trigger inverter disconnection, and the system maintained stable operation.

Table 2 presents the measured islanding detection performance of single-phase photovoltaic inverters operating under identical balanced power conditions, including the case where one inverter had its islanding protection function disabled. The results show that disconnection times varied notably—from 1.1 to 1.7 s—even within the same test scenario.

Figure 5, Figure 6, Figure 7 and Figure 8 present, for the tested inverter, the instantaneous AC voltage (U_L1SUM_), the AC-side frequency (f), and the phase 1 AC current (I_L1_). These waveforms illustrate the dynamic response of the inverters during islanding events, thereby validating the effectiveness of the implemented islanding detection mechanisms.

**Figure 9:**
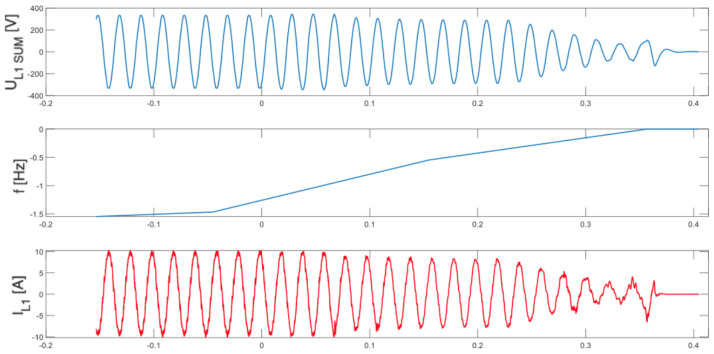
Islanding Detection of the Standalone Operation of Inverter No. 4 (Test 5).

**Figure 10:**
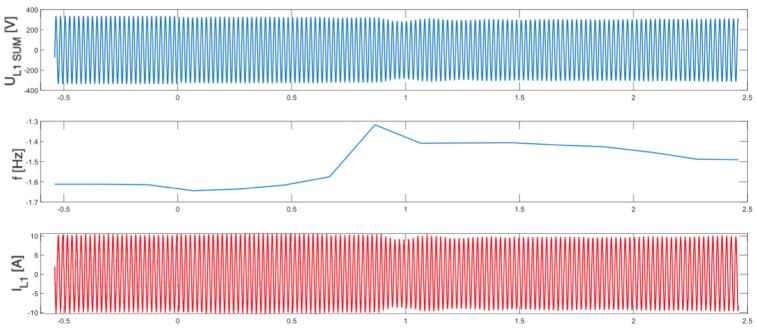
Islanding Detection of the Standalone Operation of Inverter No. 4 (Test 6).

Figure 9 and Figure 10 illustrate the performance of inverter No. 4 under unbalanced conditions, with the parameter ΔP set to +5% P_ac_ and −5% P_ac_, respectively.

In the subsequent series of tests (tests 7.1–7.3), the parallel operation of three PV inverters was examined, with inverter No. 4 present in each trial operating with its islanding detection function disabled.

**Figure 11:**
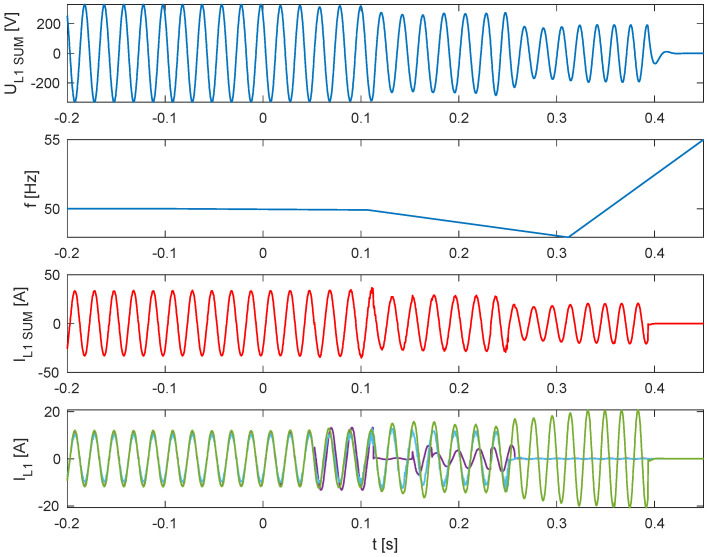
Islanding Detection of the Standalone Operation of Inverter No. 1, 2, 4 (Test 7.1).

**Figure 12:**
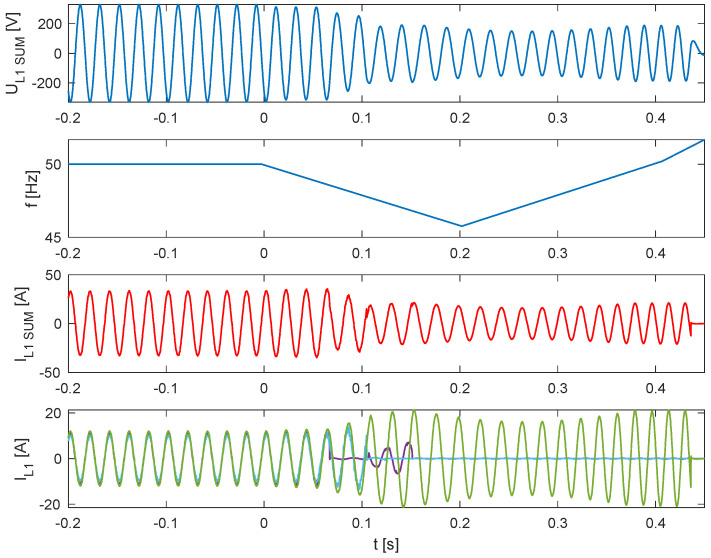
Islanding Detection of the Standalone Operation of Inverter No. 1, 3, 4 (Test 7.2).

**Figure 13:**
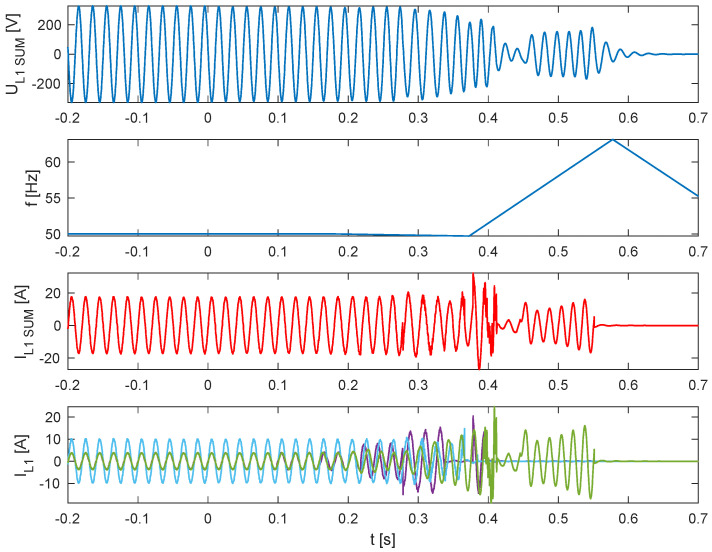
Islanding Detection of the Standalone Operation of Inverter No. 1, 3, 4 (Test 7.3).

Figure 11, Figure 12 and Figure 13 present, common for all tested three-phase inverters, the instantaneous AC voltage (U_L1SUM_), the AC-side frequency (f), the instantaneous sum of AC phase 1 currents (I_L1SUM_), and the individual phase 1 AC currents of each inverter (I_L1_). These waveforms illustrate the dynamic response of the inverters during islanding events, thereby validating the effectiveness of the implemented islanding detection mechanisms.

Analysis of the results indicates that, in the parallel operation scenario, the disconnection time was extended compared to individual operation. Recorded shutdown times ranged from 450 to 600 ms, which can be attributed to the mutual interactions of the inverters in parallel mode and changes in the dynamics of power flow in the local network. The extended response time suggests that in parallel configurations, the islanding detection characteristic is modulated depending on the cooperation of devices and the instantaneous power balance. Nevertheless, despite the prolonged response time in parallel configurations, all devices ultimately disconnected within the time limits 2 s required by the standard [3], ceasing operation in islanded mode.

**Figure 14:**
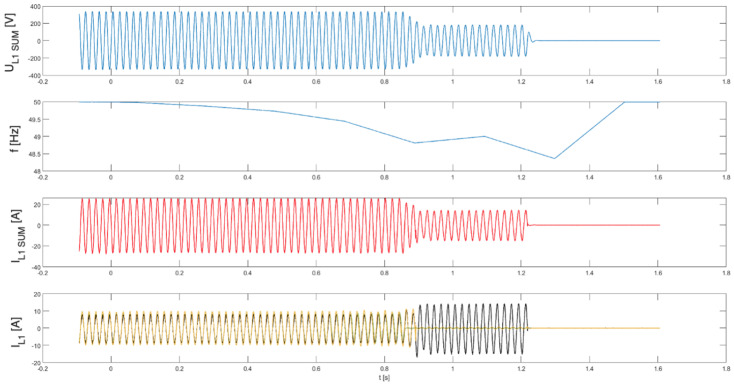
Islanding Detection of the Standalone Operation of Inverter No. 5, 6, 7 (Test 8.1).

**Figure 15:**
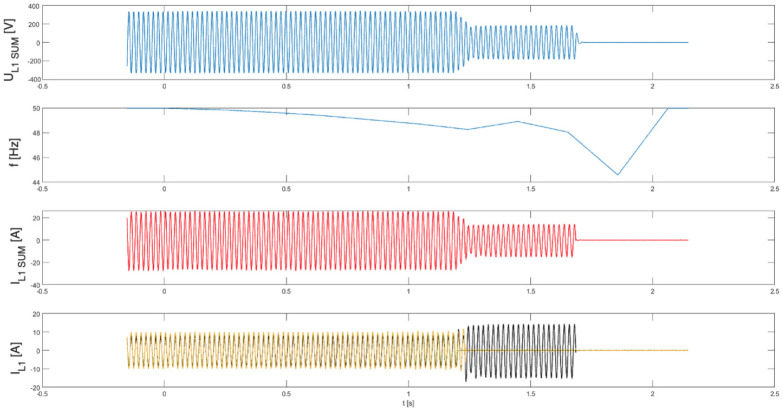
Islanding Detection of the Standalone Operation of Inverter No. 5, 6, 7 (Test 8.2).

**Figure 16:**
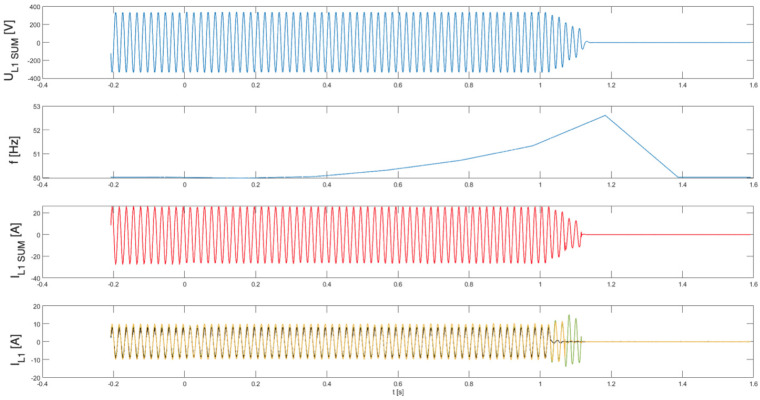
Islanding Detection of the Standalone Operation of Inverter No. 5, 6, 7 (Test 8.3).

Figure 14, Figure 15 and Figure 16 present, common for all tested single-phase inverters, the instantaneous AC voltage (U_L1SUM_), the AC-side frequency (f), the instantaneous sum of AC phase 1 currents (I_L1SUM_), and the individual phase 1 AC currents of each inverter (I_L1_). These waveforms illustrate the dynamic response of the inverters during islanding events, thereby validating the effectiveness of the implemented islanding detection mechanisms. The results show that disconnection times varied notably—from 1.1 to 1.7 s—even within the same test scenario. This observed variability has critical implications for the experiment. First, it demonstrates that the disconnection time is not a fixed property of the detection method itself, but is influenced by unit-to-unit manufacturing tolerances, slight calibration differences, or transient noise. Consequently, the experiment could not produce a single, definitive disconnection time for the scenario. Instead, its outcome is a performance range, suggesting that any single test may not be sufficient to characterize an inverter’s behavior reliably. This underscores the importance of repeated trials and statistical analysis to ensure results are representative and robust.

## 5. Extension of IEC 62116 Islanding Detection Tests to Parallel Operation of Distributed Generators

The IEC 62116 standard defines a unified test procedure for evaluating the performance of islanding detection methods in distributed generators, with particular emphasis on removing the influence of load-generation matching through the use of an RLC resonant circuit. While the standard has been effective in providing a consistent and reproducible methodology for evaluating single inverter operation, it does not explicitly address test scenarios involving multiple distributed generators operating in parallel, which have become increasingly common in modern low-voltage distribution networks. This limitation reduces the applicability of the standard under conditions typical of PV-dense feeder segments, where dynamic interactions between inverters can substantially alter the behavior of islanding detection mechanisms.

The experimental results presented in this study demonstrate that parallel operation can significantly increase the detection time of the active inverters compared to standalone tests. Although the extended detection times remained within the 2-s threshold required by grid codes, the observed increase—combined with the possibility of one unprotected inverter sustaining the island indefinitely—highlights the need for a new standardized methodology.

### 5.1. Limitations of the Current Test Procedure

IEC 62116 focuses on a worst-case NDZ created by carefully tuning the RLC load to resonate at the grid frequency. However, the standard implicitly assumes that the inverter under test is the sole active element controlling voltage and frequency in the potential island. This assumption diverges from practical LV networks, where multiple single-phase and three-phase PV inverters commonly operate simultaneously on shared feeders. In such configurations:

- Reactive and active power mismatches are not determined solely by the RLC load but distributed across multiple generation units;
- Dynamic control interactions (P–f and Q–V droop, virtual inertia, grid-support functions) modify the effective NDZ in a nonlinear manner;
- One inverter’s disturbance-injection method may be counteracted or masked by another inverter’s grid-support control;
- A unit with disabled or malfunctioning anti-islanding protection can significantly influence the detection behavior of correctly operating inverters.

These effects cannot be adequately captured by the existing single-inverter IEC 62116 test.

### 5.2. Proposed Framework for a Multi-Inverter Extension

A comprehensive extension of the existing standard should incorporate tests that evaluate islanding detection performance under controlled multi-inverter conditions. The following additions are proposed:

#### 5.2.1. Parallel-Inverter Resonant Load Test

A modified version of the classic RLC resonant load test, in which:

- The inverter under test is connected in parallel with at least one additional distributed generator to the same RLC network;
- The power ratings are scaled proportionally to maintain comparable ΔP and ΔQ conditions;
- The NDZ is evaluated for both symmetric and asymmetric power-sharing conditions.

#### 5.2.2. Fault-in-One Test

To replicate realistic worst-case scenarios, the extended standard should include a test where:

- An additional distributed generator operates with its islanding detection disabled or malfunctioning (simulated fault);
- The inverter under test is required to detect the islanding condition and disconnect within specified limits;
- The test is repeated under slight variations in load conditions to assess consistency.

This test directly reflects field conditions where protection malfunctions or incorrect configurations may occur.

#### 5.2.3. Statistical Repeatability Test

Experimental results indicate non-negligible variability in disconnection times even under identical conditions. To incorporate this observation, the standard should require:

- A minimum number of repeated trials (e.g., 10–20);
- Reporting detection time ranges rather than single representative values;
- Calculation of basic statistical descriptors (mean, standard deviation, min/max).

Such requirements would better describe real-world inverter behavior and manufacturing variance.

Overall, extending IEC 62116 to include multi-inverter scenarios would modernize the standard to reflect today’s distributed generation landscape, providing a more comprehensive assessment of islanding detection performance.

## 6. Conclusions

The rapid expansion of PV micro-installations in low-voltage networks poses new challenges for distribution system operators regarding network safety and stability. A key aspect is the detection of unintentional islanding, which protects technical personnel, connected loads, and the network itself from the adverse effects of being disconnected from the main power system.

The experimental studies assessed the effectiveness of islanding detection in selected three-phase and single-phase PV inverters from different manufacturers. Both the response time and reliability of the protection mechanisms were analyzed under conditions of negligible load-to-generation imbalance (below 1%), as well as in scenarios where the anti-islanding protection was intentionally disabled in one inverter.

The results confirmed that modern PV inverters, employing a combination of passive methods (e.g., ROCOF, ROCOV) and active methods, achieve high effectiveness in detecting islanding events. Furthermore, the parallel operation of multiple inverters provided crucial insights into their mutual interactions. An important finding from this analysis is the notable variability in disconnection times, observed for single-phase inverters—ranging from 1.1 to 1.7 s—under identical test scenarios. This variability, observed between different units and manufacturers, highlights that disconnection time is not a fixed property but is influenced by unit-to-unit tolerances and the complex interplay of multiple, simultaneously active detection algorithms.

These findings demonstrate that existing certification procedures—which primarily assess single-inverter operation—may be insufficient to fully characterize inverter behavior in realistic network environments with high PV penetration. Multi-inverter interactions significantly influence protection dynamics, potentially leading to delayed disconnection or temporary stable island formation. Therefore, the development of a supplementary test standard addressing parallel-operation conditions is essential to ensure that PV inverters maintain reliable anti-islanding performance in modern distribution networks:

- For PV inverter certification: The observed performance variability underscores that certification tests must move beyond verifying simple functionality. Standards should require statistical analysis of disconnection times across multiple units and under a wider range of grid-impedance and multi-inverter scenarios to ensure consistent and predictable performance.
- For distribution system planning and safety: The lack of a uniform response time means DSOs cannot assume all inverters in a network segment will disconnect simultaneously. This de-synchronization could lead to complex, unstable islanded conditions even when most inverters are functioning correctly. Therefore, grid protection schemes must be designed to account for this potential non-homogeneous response, potentially requiring longer safety wait-times or additional centralized protection systems.

## 7. Future Research Directions

Building on the findings of this study, several avenues for future research can further enhance the reliability and performance of islanding detection in PV inverters. Extending experimental investigations to include larger numbers of parallel-connected inverters and mixed inverter types could provide deeper insights into interaction effects and collective response times under realistic distribution network conditions. Additionally, evaluating islanding detection under rapidly fluctuating load and generation scenarios, including high penetration of intermittent renewable sources such as PV and wind, would allow assessment of algorithm robustness in dynamic operating environments.

Further development of hybrid and intelligent detection techniques, combining passive, active, and machine-learning-based approaches, could improve detection sensitivity while minimizing adverse effects on power quality. It is also important to study the interaction of advanced inverter functions, such as voltage and frequency support, reactive power optimization, and coordinated control strategies, with islanding detection mechanisms, as these may influence the overall effectiveness of protection systems.

Field validation of detection algorithms in actual low-voltage distribution networks is necessary to complement laboratory studies, capturing real-world effects such as communication delays, harmonics, and network topology changes. Additionally, there is a need to update certification and testing protocols to better reflect multi-inverter interactions and dynamic operating conditions, which could inform revisions to standards [2,3]. Finally, the impact of emerging technologies, including battery energy storage, electric vehicles, and microgrid controllers, on islanding detection performance warrants investigation to ensure safe and reliable integration of distributed generation in increasingly complex low-voltage networks.

## Figures and Tables

**Table 1:** Cumulative Results of Islanding Detection Time Tests for Three-Phase PV Inverters.

Test Number	Connected Devices	Islanding Detection Protection Status (On/Off)	Generated Active Power P_AC_	Power Imbalance	Disconnection Time t_off_ [s]
1	Inverter 1	On	5 kW	ΔP < 1% P_AC_ ΔQ < 1% P_AC_	0.15
2	Inverter 2	On			1.95
3	Inverter 3	On			0.10
4	Inverter 4	Off			>25
5	Inverter 4	Off		ΔP = 5% P_AC_	0.36
6	Inverter 4	Off		ΔP = −5% P_AC_	>2.5
7.1	Inverters: 1, 2, 4	2 × On, 1 × Off	15 kW	ΔP < 1% P_AC_ ΔQ < 1% P_AC_	0.45
7.2	Inverters: 1, 3, 4	2 × On, 1 × Off			0.55
7.3	Inverters: 2, 3, 4	2 × On, 1 × Off			0.15

**Table 2:** Cumulative Results of Islanding Detection Time Tests for Single-Phase PV Inverters.

Test Number	Connected Devices	Islanding Detection Protection Status (On/Off)	Generated Active Power P_AC_	Power Imbalance	Disconnection Time t_off_ [s]
8.1	Inverters: 5, 6, 7	2 × On, 1 × Off	~10 kW	ΔP < 1% P_AC_ ΔQ < 1% P_AC_	1.2
8.2	Inverters: 5, 6, 7	2 × On, 1 × Off			1.7
8.3	Inverters: 5, 6, 7	2 × On, 1 × Off			1.1

## Data Availability

Restrictions apply to the availability of these data.

## Abbreviations

The following abbreviations are used in this manuscript:

AC	Alternating current
AFD	Active frequency drift
DC	Direct current
DER	Distributed energy resource
DSO	Distribution system operator
EMT	Electromagnetic transient
HIL	Hardware-in-the-loop
LV	Low voltage
LFSM-O	Limited frequency sensitive mode—overfrequency
NC RfG	Network code for all generators
OVRT	Overvoltage ride through
PCC	Point of common coupling
PV	Photovoltaic
PMU	Phasor measurement unit
ROCOF	Rate of change of frequency
ROCOV	Rate of change of voltage
RMS	Root mean square
SFS	Sandia frequency shift
SVS	Sandia voltage shift
THD	Total harmonic distortion
UVRT	Undervoltage ride through
